# Bone density and body composition in newly licenced professional jockeys

**DOI:** 10.1007/s00198-017-4086-0

**Published:** 2017-06-13

**Authors:** K. A. Jackson, M. T. Sanchez-Santos, A. L. MacKinnon, A. Turner, K. Kuznik, S. Ellis, C. Box, J. Hill, M. K. Javaid, C. Cooper, N. K. Arden, J. L. Newton

**Affiliations:** 1 Arthritis Research UK Centre for Sport, Exercise and Osteoarthritis, Nuffield Department of Orthopaedics, Rheumatology and Musculoskeletal Sciences, University of Oxford, Oxford, UK; 2 Oxford NIHR Musculoskeletal Biomedical Research Unit, Nuffield Department of Orthopaedics, Rheumatology and Musculoskeletal Sciences, University of Oxford, Oxford, UK; 3 British Horseracing Authority, 75 High Holborn, London, UK; 4 University of Oxford Medical School, Oxford, UK; 5 MRC Lifecourse Epidemiology Unit, Southampton General Hospital, University of Southampton, Southampton, UK

**Keywords:** Athlete, Body composition, Bone density, DEXA, Jockey

## Abstract

**Summary:**

The primary objective is the description of bone mineral density (BMD) and body composition in newly licensed jockeys. One in three male, flat jockeys has a very low bone mineral density. Further research is needed to assess the short-term risk of fractures and long-term health implications of these findings.

**Introduction:**

Describe bone mineral density (BMD) and body composition in entry-level male and female, flat and jump jockeys in Great Britain.

**Methods:**

Data was collected on jockeys applying for a professional jockey license between 2013 and 2015. Areal BMD at the spine, femoral neck (FN), total hip and body composition were assessed by dual-energy X-ray absorptiometry (DXA) scan. We examined differences between BMD and body composition by gender and race type (flat or jump). Volumetric bone mineral apparent density (BMAD) of the spine and FN was also calculated to account for group differences in bone size.

**Results:**

Seventy-nine male flat jockeys (age 18.5 ± 1.9, BMI 19.0 ± 1.4), 69 male jump (age 20.7 ± 2.0, BMI 20.6 ± 1.3) and 37 female flat jockeys (age 19.3 ± 2.0, BMI 20.8 ± 1.7) took part in this study. Spine BMD Z-scores ≤−2 for male flat, male jump and female flat jockeys were 29, 13 and 2.7%, respectively. Spine BMD was lower in male than female flat jockeys (*p*<0.001). All BMD scores were lower in male flat compared to male jump jockeys (*p*<0.001). Body fat percent (BF %) was lower in male flat jockeys compared to male jump and female flat jockeys (*p*<0.05). Lean mass index (LMI) was lower in male flat compared to male jump jockeys (*p*<0.001).

**Conclusions:**

Male flat jockeys had a significantly lower BMD, LMI and BF% compared to jump jockeys and female flat jockeys. Male flat jockeys had lower spine BMD scores than females. Individual bone maturation may influence these findings. Further investigation into the relevance of low BMD and altered body composition on jockey health is required.

**Electronic supplementary material:**

The online version of this article (doi:10.1007/s00198-017-4086-0) contains supplementary material, which is available to authorized users.

## Introduction

Professional horse racing is a popular sport in the UK. It is divided into flat racing and jump (National Hunt) racing. Flat racing involves distances of 5–20 furlongs (1 furlong = 0.201 km), whereas jump racing covers longer distances (16–36 furlongs) with horses jumping over a minimum of eight obstacles. In the UK, there are currently over 400 licenced professional jockeys, over 350 amateur jockeys and over 5000 stable staff.

Newly licenced jockeys are an important sub-group of jockeys to study. In this weight-restricted sport, unhealthy weight loss strategies are used by jockeys to make weight for a race, [1, 2]. This may have negative effects on jockey health [3, 4]. Less-experienced jockeys are under further pressure to make lower weights as a result of being able to carry a ‘claim’. Currently, in the UK, flat jockeys have a minimum riding weight of 8 stone (50.8 kg). However, when first licenced, they can claim a weight allowance which allows them to race-ride 7 lbs lighter, i.e. 7 stone 7 lbs (47.6 kg). This claim reduces as the jockey wins races and ceased altogether once they have 95 winners. Race riding at a lower weight than more experienced riders is felt to help compensate for their inexperience.

Newly licenced jockeys are young (between the ages of 16–25 years) and should have achieved 90% of peak bone mass by their early 20’s. Therefore, lifestyle choices in the newly licenced jockey population is key for their immediate, but also future, bone health [5].

Several small studies have identified low bone mineral density (BMD) in the male jockey population. Despite the limitations of small sample sizes and potential recruitment bias, these studies suggest a worrying trend [6–10]. Female jockeys seem to be at less risk of low BMD although current evidence for this is limited [11]. Low BMD is known to be associated with an increased risk of both traumatic and low-impact fractures in older populations [12]. There is an assumption that this would be the same in a younger population, but evidence for increased traumatic fracture risk with low BMD is lacking. Jockeys fall off their horses at speed and from a height on a relatively frequent basis and thus are at risk of traumatic injury. Jump jockeys fall more often but have less injuries per fall (UK data 68 falls per 1000 rides; 180 injuries per 1000 falls); whereas, flat jockeys fall less often, but are more likely to injure themselves (UK data 4.4 falls per 1000 rides; 398 injuries per 1000 falls) [13]. Bone fractures are one of the commonest injuries sustained [14].

The significance of body composition for injury and health in jockeys is not currently clear. Previous studies have observed lower body fat [2, 6, 9, 11] and lean mass in jockeys [6, 11] when compared to the normal population. Epidemiological studies have shown lean mass (LM) to have a significant positive correlation with BMD [15, 16]. Fat mass (FM) appears to have weaker positive correlation to BMD, and this relationship is affected by age, gender and menopausal status [15]. If the weight-bearing effect of the FM is adjusted for, a higher FM may in fact have an inverse association with BMD [17]. Underlying muscle weakness has been postulated as a contributing factor to the high rates of falls and subsequent injury in racing [18].

Current literature suggests a significant problem with bone health in this population, and our aim is to examine bone health and body composition parameters in a larger, more representative cohort of male and female jockeys. The primary objective is to describe whole body BMD in newly licenced professional jockeys with sub-analysis by gender and race type. The secondary objective is to describe body composition in newly licenced professional jockeys with sub-analysis of the same factors.

## Methods

### Subjects

All entry-level professional jockeys attending the professional jockey licencing courses between January 2013 and November 2015 were invited to have a DEXA scan performed as part of their medical screening. There were 216 newly licenced jockeys reported by the PJA in this time frame. One hundred and eighty-six flat and jump jockeys (86%) took part in the screening programme. The screening involved a routine DEXA scan performed at the time of initial licence. Data was collected by the medical department at the British Horse Racing Authority (BHA) and by the Professional Jockeys Association Medical Adviser (PJAMA). The data was anonymised before being exported for analysis by the study team.

### Ethics approval

Ethics approval (MS-IDREC-CX-2014-147) was obtained for this study from the Central University Ethics Committee (CUREC).

### Assessment of bone and body composition

Bone mineral content (BMC), bone area (BA) and areal BMD at the spine (L1–L4), femoral neck (FN) and total hip as well as body composition (FM and LM (excluding bone mass)) were assessed by DXA scan using two Hologic Discovery densitometers (*n* = 146 and *n* = 3 images, respectively), and one mobile Hologic Explorer (*n* = 37 images).

The BMD (g/cm^2^) Z-score (number of standard deviations (SD) more or less than a same age group and sex reference mean) at the spine, FN or total hip of ≤ − 1 and ≤ − 2 was used to sub-group participants, [19, 20]. ‘National Health and Nutrition Examination Survey (NHANES) 2008’ reference database was used [21].

Areal BMD tends to underestimate true BMD in small, thin individuals. Therefore, the best way to eliminate the effect of bone size on BMD in this young population was to calculate the volumetric bone density, or bone mineral apparent (BMAD) (g/cm^3^) [22]. The BMAD values [22, 23] were calculated for the lumbar spine and FN as follows:

- Spine BMAD = BMC of spine/area^3/2^
- FN BMAD = BMC of FN/area^2^

A comparison study on BMD measurements between the two Hologic Discovery scanners using a European-standard phantom spine was performed. Ten phantom samples with the same characteristics were entered in each instrument software programme. Bland Altman plots and coefficient of variations (CV) of the scanners were calculated, and they showed very good levels of agreement and good machine precision (CV% <1.2%). (Text A.1). The Explorer Hologic machine was dismantled, so we were not able to include it in the comparison study.

Height and body mass were measured following standardised procedures before the DEXA scan was performed. Body weight was measured with a set of Seca electronic weighing scales and body height was measured standing using a height measure. Body mass index (BMI) was calculated as the ratio of the weight to the square of height in meters (kg/m^2^). It was categorised according to the World Health Organization (WHO) classification: underweight (BMI <18.5 kg/m^2^), normal weight (18.5 ≤BMI ≤ 24.9 kg/m^2^) and overweight/obesity (BMI ≥25 kg/m^2^)), and was also used as a continuous variable in this analysis. The two suggested height-normalised indices for body composition, LM index (LMI) and FM index (FMI) was calculated as LM/height squared (kg/m^2^) and FM/height squared (kg/m^2^), respectively. Body fat percentage (BF %) was assessed as FM (kg) as a percentage of total body weight (kg).

### Statistical analysis

All analyses were conducted using the Stata version 13.1 statistical software (StataCorp, College Station, TX). The distribution of bone density and body composition were analysed by gender and race type (flat or jumps). There was only one female jump jockey who was therefore excluded from the analysis. Subject characteristics, bone measurements and body composition measurements were summarised by mean and SD or medians and percentiles for continuous variables and frequency (percentage) for categorical variables. Differences between groups (male flat jockeys vs. male jump/female flat jockeys) were identified using an independent samples *t* test or Mann–Whitney *U* test depending on data distribution and chi-squared if the variables were categorical.

To examine whether the different scanner may contribute to differences between groups, analyses were repeated excluding jockeys measured by the Hologic Explorer scan (*n* = 37). No substantial differences in the results were observed (Table A.2.1).

The level of significance was set at *p* < 0.05 for all statistical analysis.

## Results

### Descriptive data

Descriptive data and anthropometric characteristics are presented in Table 1. Forty-two percent of the participants were male flat jockeys, 37.1% male jump jockeys and 19.9% were female flat jockeys. The mean ± SD age of male jump jockeys (20.7 ± 2.0 years old) and female flat jockeys (19.3 ± 2.0) was significantly higher than that of male flat jockeys (18.5 ± 1.9), *p* < 0.001 and *p* = 0.03, respectively. There were significant differences in weight and height between groups. Male flat jockeys were shorter and lighter than male jump jockeys, but they were taller and heavier than the female, flat jockeys (1.67 vs. 1.76/1.57 m and 52.9 vs. 63.7/51.6 kg, respectively) (Table 1). Differences between the groups also reached significance for FM and LM. Male flat jockeys had a smaller LM and FM than the male jump group and smaller FM values than the female flat jockey group. In contrast, male flat jockeys had greater LM than that of the female flat jockeys.

**Table 1:** Jockey characteristics according to gender and type of racing

	Male		Female	Flat-male vs. flat-female	Flat-male vs. jumps-male
	Flat	Jump	Flat	*p* value	*p* value
*N* (%)	79 (42.5)	69 (37.1)	37 (19.9)		
Age (years)	18.5 ± 1.9	20.7 ± 2.0	19.3 ± 2.0	0.03	<0.001
Weight (kg)	52.9 ± 2.9	63.7 ± 3.6	51.6 ± 4.0	0.04	<0.001
Height (m)	1.67 ± 0.06	1.76 ± 0.05	1.57 ± 0.05	<0.001	<0.001
LM (kg)	42.5 ± 2.6	50.7 ± 3.1	36.4 ± 2.7	<0.001	<0.001
FM (kg)	7.6 ± 1.3	9.9 ± 1.7	12.4 ± 2.4	<0.001	<0.001

Values are mean ± SD except where noted.

*LM* lean mass, *FM* fat mass

### Bone mass

All measurements of bone mass are shown in Table 2. The BMC and BMD at all skeletal sites were significantly lower in male flat jockeys than in male jump jockeys (Table 2). Male flat jockeys also had lower BMC at the spine than the female flat jockeys, but significantly higher BMC values at the FN and at total hip. Differences between BMD at the spine and at total hip were found in male and female flat jockeys, being significantly lower in male; however, no differences between BMD at FN were found.

**Table 2:** Bone mass information according to gender and type of racing

	Male		Female	Flat-male vs. flat-female	Flat-male vs. jumps-male
	Flat	Jump	Flat	*p* value	*p* value
Spine BMC (g)	51.85 ± 7.27	65.26 ± 9.04	54.71 ± 6.23	<0.05	<0.001
Spine BMD (g/cm^2^)	0.876 ± 0.091	0.969 ± 0.087	0.987 ± 0.083	<0.001	<0.001
Spine BMAD (g/cm^3^)	0.114 ± 0.011	0.118 ± 0.011	0.132 ± 0.013	<0.001	<0.05
Z-score ≤ −1.0, *n* (%)	60 (76.0)	36 (52.2)	6 (16.2)	<0.001	<0.001
Z-score ≤ −2.0, *n* (%)	23 (29.1)	9 (13.0)	1 (2.7)	<0.001	0.01
FN BMC (g)	4.43 ± 0.38	5.26 ± 0.69	4.06 ± 0.48	<0.001	<0.001
FN BMD (g/cm^2^)	0.831 ± 0.070	0.935 ± 0.093	0.840 ± 0.077	0.534	<0.001
FN BMAD (g/cm^3^)	0.155 ± 0.113	0.168 ± 0.023	0.176 ± 0.023	<0.001	<0.001
Z-score ≤ −1.0, *n* (%)	27 (34.2)	6 (8.7)	3 (8.1)	0.002	<0.001
Z-score ≤ −2.0, *n* (%)	1 (1.3)	–	–	–	–
Total hip BMC (g)	35.01 ± 3.84	43.18 ± 6.25	30.22 ± 4.03	<0.001	<0.001
Total hip BMD (g/cm^2^)	0.924 ± 0.084	1.017 ± 0.100	0.959 ± 0.075	0.04	<0.001
Z-score ≤ −1.0, *n* (%)	33 (41.8)	7 (10.1)	1 (2.7)	<0.001	<0.001
Z-score ≤ −2.0, *n* (%)	–	–	–	–	–

Values are mean ± SD except where noted. Six jockeys did not have data available for BMD Z-score at the spine and five jockeys did not have available BMD Z-score at neither FN or at total hip. Seven jockeys did not have data available for BMC at all skeletal sites

*BMC* bone mineral content, *BMD* bone mineral density, *BMAD* bone mineral apparent density, *FN* femoral neck

Male flat jockeys had significantly lower bone density of the spine, FN and at the hip than male jump jockeys and female flat jockeys.

Both lumbar and FN BMAD values were significantly lower in male flat jockeys than in male jump jockeys and female flat jockeys, indicating that the actual density differences between groups are independent of differences in bone size.

### Body composition

**Fig. 1:**
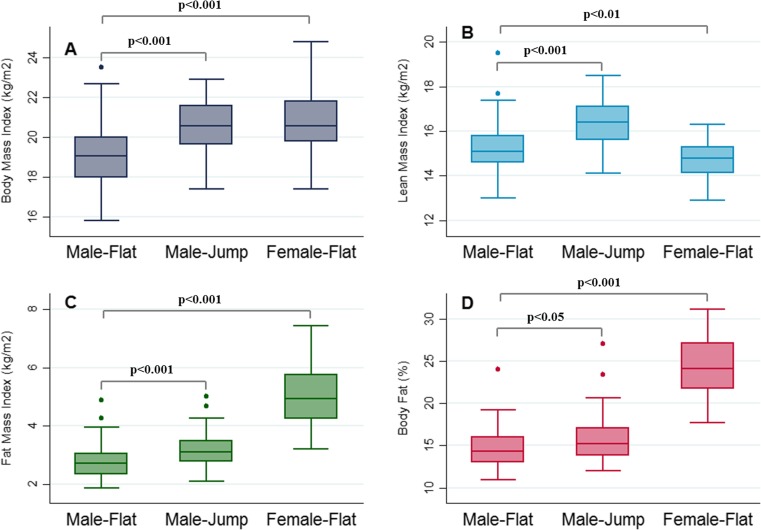
Box plots of **a** BMI (kg/m^2^), **b** LMI (kg/m^2^), **c** FMI and **d** BF (%) according to gender and type of racing. *BMI* body mass index, *LMI* lean mass index, *FMI* fat mass index, *BF* body fat

There were significant differences in body composition by gender and type of racing (Fig. 1). The mean BMI ± SD was 19.0 ± 1.4, 20.6 ± 1.3 and 20.8 ± 1.7 kg/m^2^ in male flat, male jump and female flat jockeys, respectively. Underweight was present in 39.2% of male flat, 4.4% of male jump and 10.8% of female flat jockeys. No jockeys presented overweight/obese. LMI, FMI and BF% were significantly lower in male flat jockeys compared with male jump jockeys (LMI 15.3 ± 1.1 vs. 16.4 ± 1.1 kg/m^2^; FMI 2.7 ± 0.5 vs. 3.2 ± 0.6 kg/m^2^ and BF% 14.6 ± 2.3 vs. 15.7 ± 2.7). Male flat jockeys had significantly smaller value FMI and BF% compared to female flat jockeys (24.4 ± 3.7). In contrast, male flat jockeys had significantly higher values of LMI than that of the female flat jockeys (14.7 ± 0.9 kg/m^2^).

## Discussion

The results from this study give us a picture of current bone health and body composition in a large group of newly licenced professional jockeys. To date, in the published literature, studies of bone health and body composition in professional jockeys have been relatively small, typically between 10 and 20, predominantly male jockeys. Male jockeys have been shown to have lower bone density than the normal population [6–9, 11], lower body fat [2, 6, 8, 9, 11] and lower lean mass [6, 11]. One of the few studies to include female jockeys reported normal bone density [11] whilst another study showed two female jockeys (*n* = 5) were osteopenic, [8].

Our study is a large and generalisable representation of newly licenced jockeys confirming previous findings of low BMD in male jockeys whilst extending our understanding of bone health in female jockeys and body composition values in all jockeys. We found that 76% of male, flat jockeys have a low spine BMD (less than one SD below the average in the age-sex-reference) and 29% had a very low BMD (less than two SD below the average age-sex-reference [20]), as opposed to the expected 16 and 2.5%, respectively. However, these observations were not limited to the flat-male jockeys with 52% of jump jockeys also having a low BMD at the spine. Female jockeys had normal BMD at all sites. We observed that male flat jockeys had a significantly lower BMD in their spines than female flat jockeys. This observed difference is opposite to the general population where women have a lower spinal BMD than men. This reversal has been observed before in a small (*n* = 16) voluntary group of male and female flat jockeys, [11]. This larger generalisable cohort confirms these findings.

The factors affecting bone health in jockeys have been the subject of several studies. Intrinsic factors are centred around poor nutrition with a reduced calorie intake leading to a state of energy deficiency [2, 3, 7–9, 11, 24]. The resulting low energy availability is thought to affect the hormonal profile [7] and negatively affect metabolic processes including the rate of bone protein synthesis and bone mineralisation, [3, 9, 11]. Reduced calcium intake and low vitamin D may also contribute although short-term supplementation has not shown to affect bone material properties in young jockeys [25–27]. Extrinsic factors may impact bone health such as smoking or the amount of weight-bearing exercise performed. Dolan et al. found that 38% of jockeys in their study smoked and over half admitted to using it as a form of weight control [2]. The efforts of flat jockeys to maintain a very low minimum weight in order to race is leading to poor bone health through a combination of the above factors. Our observed differences in BMD between flat and jump jockeys is likely the result of the higher minimum race-riding weight of 10 stone (63.5 kg) for jump jockeys, compared to 8 stone (50.8 kg) for flat jockeys. The observed differences between male and female jockeys can also be explained by their shorter stature allowing them to make weight without resorting to such severe or persistent weight loss strategies [11]. Poor bone health in male jockeys is happening at a time when these young men are reaching peak bone mass. A large longitudinal study has demonstrated differing ages for peak bone mass (PBM) between sexes and at different sites [28]. Male total hip and femoral neck PBM was achieved between 19 and 22 years of age and 17–19 years of age respectively and male lumbar spine PBM occurred between 19 and 33 years.

Body composition and demographic results confirm previous findings of lighter, smaller flat jockeys compared to jump jockeys. It also confirms the finding of lower LM in jockeys. The average BMI in all three groups of jockeys was predominantly in the lower half of normal range (normal BMI 18.5–25), running into underweight particularly in the male flat jockeys. BF% was only slightly lower in all jockey groups than the general population. The BF% of male flat, male jump and female flat jockeys were 14.6 ± 2.25, 15.67 ± 2.68 and 24.35 ± 3.68, respectively, which is lower than that of 18 to 34-year-old males and females from the general population (17.9 ± 4.7 and 28.7 ± 6.4, respectively) [29]. The BF% of flat, male jockeys in this larger cohort is at odds with previous smaller studies of male, flat jockeys where BF% values were found to be much lower [2, 6, 9, 11]. FMI (accounting for height) in male flat jockeys in our study was 2.7 ± 0.5 kg/m^2^. A previous small study in male, flat jockeys reported FMI as significantly lower (1.65 ± 0.62) [6]. This discrepancy in FM raises the question of recruitment bias in these small studies where volunteers may already be interested in their body composition and therefore actively maintaining lower BF than their peers who did not volunteer. One study of flat, male professional jockeys (*n* = 19) found BF% of 13 ± 3.0 which is nearer the findings of our larger cohort, but this cohort of jockeys was considerably older (27 ± 5 years) than our newly licenced group. It is likely they are riding at higher weights as they will no longer be carrying a claim. There are a number of studies looking at BF % (means) in other weight-restricted and lightweight sports: male lightweight rowers 8.5%, *n* = 120, mean age 21 years [30]; wrestling 8.3%, *n* = 35, mean age 24.3 years [30]; female sub-elite cyclists 16%, *n* = 76, mean age 23.8 years [31]; male competitive club cyclists 9.9%, *n* = 14, mean age 28.1 years [32] and male boxers 9.76%, *n* = 14, mean age 21 years [6]. BF% in these studies is significantly lower than for our jockey cohort.

Population based studies of LMI in men have reported higher values than in our male jockey population. NHANES data of white male age range 20–25 (*n* = 235) had an average LMI of 19.0 and white, 20 to 25-year-old females (*n* = 323) had an average LMI of 16.0, [21]. The Hertfordshire cohort study reported LMI in men of 17.82 ± 1.44 and women of 15.06 ± 1.55, [33]. A small study of jockeys used physically active male, age- and BMI-matched controls and the controls had LMI of 18.2 ± 1.51 [7]. In the same study, LMI is reported in professional jockeys as 18.33 ± 1.46. Another small study of flat jockeys as 18.19 ± 1.3(*n* = 14) and in jump jockeys as 18.125 ± 1.23(*n* = 16). Several studies look at LM (kg), but direct comparison of LM without correcting for height difference between sports makes this difficult to interpret. One small study found the LMI in boxers (*n* = 14) to be 18.25 ± 1.23 [6]. The male, flat and jump jockeys had significantly lower LMI (15.3 ± 1.1 and 16.4 ± 1.1 kg/m^2^, respectively) than all the groups studied above. As we do not know the maturation status of our jockeys, there may have been a number of the youngest jockeys who had not yet fully physically matured. However, using an age-specific stratified analysis according to time of expected peak LMI (<19 vs. ≥19 years) [28], both groups still exhibited a low LMI which suggests there may be other factors involved (Table A.2.2).

These observed differences in body composition may be related to the weight loss practices in the jockey population. Cyclical daily efforts to make weight for each race-ride may be having a detrimental effect on metabolism with loss of lean mass instead of fat mass. In addition, some jockeys may be intentionally avoiding types of exercise that they believe could increase muscle mass as part of their weight control strategy. Time pressure from extensive travelling between races also reduces the chance of these young jockeys doing any dedicated fitness training.

### Strengths and limitations

The strengths of this study are that it is the largest cross-sectional study of male and female jockey BMD and body composition. With almost full recruitment, it is highly generalisable. All the jockeys were newly licenced, increasing the homogeneity of the cohort.

The limitations of this study are that the DEXA scans were performed on three different scanners (all Hologic scanners). A phantom spine study was performed to observe any differences between the two Hologic Discovery scanners. The differences in BMD were clinically insignificant (Appendix A.1). The Hologic Explorer machine had been dismantled so we were unable to include it in comparison study. However, we performed a sensitivity analysis excluding jockeys measured by the Explorer scan and found no significant impact on the results and interpretation of this study (Table A.2.1). The other important limitation of this study was the non-availability of any indicator of maturation; therefore, we could not determine whether there was a high proportion of late maturers in this population. We conducted an age-specific stratified analysis according to time of expected peak BMD (younger <21 year, and older 21 years or older). The percentage of BMD Z-score < −2.0 in both age groups was significantly higher than expected for their chronological age (Table A.2.3).

## Conclusion

This study in a large, representative group of professional, entry-level jockeys has confirmed that 29% of newly licenced male, flat jockeys have a very low BMD (Z-score ≤ −2) in their spines. This is occurring at an age when these young jockeys are reaching their peak bone mass, which strongly influences future bone health and fracture risk. What is currently less clear is how less-than-optimal bone health influences immediate fracture risk in these young jockeys. The male, flat jockeys have a significantly lower BMD at the spine than female flat jockeys, which is the reverse of the normal population. This study also identified that flat jockeys appear to have a proportional lower LM than would be expected in both the normal population and other low-weight sports. The study cohort was young and so maturation may contribute to these findings. This study provides robust evidence of poor male jockey bone health to support previous findings as well as body composition data on a large jockey cohort. We would recommend further research establishing the relationship between poor bone health in newly licenced jockeys and subsequent injury in this population, as well as the relationship between body composition and rates of falls and injury.

## Electronic supplementary material

Esm 1 (DOCX 2587 kb).

Esm 2 (DOCX 18 kb).
