# Supplementary material for: Bone density and body composition in newly licenced professional jockeys
Source: Osteoporos Int. 2017 Jun 13;28(9):2675–82. doi: 10.1007/s00198-017-4086-0 (PMC5550520; doi:10.1007/s00198-017-4086-0)
Supplement: Supplementary file 1 [file 198_2017_4086_MOESM1_ESM.docx]

**TEXT A.1**

**Hypothesis/Goal**

The purpose of this study was to compare Bone Mineral Density (BMD) measurements by using two Hologic instruments.

**Methods**

BMD values of L1, L2, L3 and L4 were measured using two Hologic Discovery instruments (Anglian Medical Musculoskeletal (scan 1) and FastTrack (scan 2)). Ten phantoms samples with the same characteristics: weight=50 kg, height=157 cm, Male, white and age between 21 and 22 years old were entered in each instrument software program.

**Statistical analysis**

Data were expressed as mean± standard deviation (SD). Comparisons between the values of the two scanners were performed using paired t tests. Degree of agreement between two instruments was assessed according to Bland and Altman method (1). Bland–Altman plots were constructed using Stata with the difference between the two instruments (bias) plotted against the mean. The mean was plotted on the x-axis in order to avoid regression towards the mean (2). Limits of agreement (LoA) were defined as “bias ± 1.96 standard deviation (SD) of the bias” (1). These values define the range within which most differences between measurements by the two instruments are expected to lie. Linear regression analysis was conducted to study the relationship between average (as independent variable) and difference (as dependent variable) of the scanners measurements. Percentage difference was calculated as difference divided by the average measured by both scanners. We would consider that instruments have good agreement if the percentage difference mean BMD values and their 95% confidence intervals (95%CI) don’t exceed 1%, being this percentage the smallest clinically acceptable difference (SDD) between both instruments. Last, DXA precision for each machine was determinate by the coefficient of variation (CV %)

**Results**

The mean and SD of BMD for each vertebra (L1, L2, L3 and L4) for the two Hologic scanners are presented in Table A1.1. Values of BMD vertebra 1, 2 and 3, on average, were very similar between the two instruments. In contrast, Hologic scan 2 gave significantly higher values for BMD at L4, on average, compared to Hologic scan 1 (p<0.01).

Bland and Altman plots for agreement between the two instruments are shown in Figure A1.1. The solid horizontal line represented the average difference and the dashed horizontal lines represent the 95% confidence limits (LoA). All measurements were within the 95% confidence interval and the line of equality (0) was within the CI of the mean difference, except for L4 BMD. These results suggest that there was good agreement between the two instruments. However, differences regressed by means of both instruments revealed significant regression coefficients for BMD for L1, L3 and L4 (p<0.01). It means that a trend in the bias was found, the bias increase as the absolute value of BMD increased. Hence, the methods did not agree equally through the range. The mean estimate averaged of BMD for L1 and L2 for Hologic scan 1 were 0.002 and 0.004 g/cm^2^, respectively, higher than the mean estimate for Hologic scan 2. In contrast, for L3 and L4 BMD, the mean estimate for the Hologic scan 1 were lower by 0.004 and 0.010 g/cm^2^, respectively, than mean estimate for the Hologic scan 2 (Figure A1.1).

The percentage differences of the scanners measurements are presented in Table A1.2. On average, the percentage differences for BMD lumbar spine L1, L2 and L3 are lower than 1% (-0.18%, -0.39% and 0.45%, respectively), except vertebra L4, with an average of 1.27%.

The coefficients of variation of the spine BMD L1-L4 varied between 0.4% and 1.1 %. The greatest CV % was found for L4 by scan 2 (1.16%).

**Conclusions**

- No significantly difference on average of BMD at vertebra 1, 2 and 3 between the two instruments was found. In contrast, a differences in mean between scanners was found for BMD at L4
- There was a good agreement between the two instruments, however, systematic bias were found for L1, L3 and L4
- On average, the percentage differences for BMD lumbar spine L1, L2 and L3 are lower than 1% (the smallest clinically acceptable difference), however L4 had a percentage error (1.3%) slightly higher
- In future analysis, we will use both scanners together, although we will check if the effect of BMD with the outcome differs by scanners for making decision.

**Table A1.1.** Comparison of BMD for each vertebra derived by the two Hologic scanners

| **BMD** | **Anglian (Scan 1)** | **FastTrack (Scan 2)** | **p-value** |
| --- | --- | --- | --- |
| **L1 (g/cm2)** | 1.238±0.004 | 1.235±0.013 | 0.634 |
| **L2 (g/cm2)** | 1.086±0.005 | 1.082±0.006 | 0.053 |
| **L3 (g/cm2)** | 0.930±0.003 | 0.934±0.009 | 0.138 |
| **L4 (g/cm2)** | 0.770±0.004 | 0.780±0.009 | **0.033** |

Note: Values are mean ± standard deviation. Means of the both instruments were compared by using the paired t-test.

BMD, bone mineral density

**Table A1.2.** % difference between BMD by the two Hologic scanners.

| **BMD** | **%Differences^a^** | **95% CI** |
| --- | --- | --- |
| **L1 (g/cm^2^)** | -0.18% | -1.00% to +0.64% |
| **L2 (g/cm2)** | -0.39% | -0.78% to +0.01% |
| **L3 (g/cm2)** | 0.45% | -0.18% to +1.07% |
| **L4 (g/cm2)** | 1.27% | +0.13% to +2.41% |

BMD, bone mineral density; CI, confidence of interval

^a^%Difference = ((Hologic scan 2 – Hologic scan 1)/Mean)*100

**Figure A1.1.** Bland-Altman plots of BMD for each vertebra (L1, L2, L3 and L4) measured by the two Hologic instruments. The differences between Hologic scan 2 and 1 were plotted against the mean values measured by the two instruments. The lines and dashed lines are mean and 95% limits of agreement (±1.96 standard deviation), respectively.

**References**

1.	Bland JM, Altman DG. Statistical methods for assessing agreement between two methods of clinical measurement. Lancet. 1986 Feb 8;1(8476):307-10.

2.	Bland JM, Altman DG. Comparing methods of measurement: why plotting difference against standard method is misleading. Lancet. 1995 Oct 21;346(8982):1085-7.
