# Supplementary material for: Bone density and body composition in newly licenced professional jockeys
Source: Osteoporos Int. 2017 Jun 13;28(9):2675–82. doi: 10.1007/s00198-017-4086-0 (PMC5550520; doi:10.1007/s00198-017-4086-0)
Supplement: Supplementary file 2 [file 198_2017_4086_MOESM2_ESM.docx]

**Table A2.1.** Bone mineral density information according to gender and type of racing (excluding images from Hologic explorer scan (n=37))

|  | Male |  | Female |
| --- | --- | --- | --- |
|  | Flat | Jump | Flat |
| Spine BMD (g/cm^2^) | 0.859 ± 0.077 | 0.962 ± 0.082 | 0.986 ± 0.088 |
| Z-Score ≤ -1.0, n (%) | 46 (80.7) | 31 (55.4) | 6 (20.0) |
| Z-Score ≤ -2.0, n (%) | 21 (36.8) | 7 (12.5) | 1 (3.3) |
| FN BMD, (g/cm^2^) | 0.826 ± 0.066 | 0.928 ± 0.096 | 0.847 ± 0.081 |
| Z-Score ≤ -1.0, n (%) | 20 (35.1) | 6 (10.7) | 3 (10.0) |
| Z-Score ≤ -2.0, n (%) | 1 (1.8) | - | - |
| Total Hip BMD (g/cm^2^) | 0.919 ± 0.079 | 1.009 ± 0.098 | 0.969 ± 0.075 |
| Z-Score ≤ -1.0, n (%) | 25 (43.9) | 6 (10.7) | 1 (3.3) |

FN, femoral neck; BMD, Bone Mineral Density.

Values are mean ± SD except where noted.

**Note:** There were no differences statistically significant between BMD at all skeletal sites by Hologic discovery (n=149) and Hologic explorer (n=37)

**Table A2.2.** Bone mineral density information according to age, gender and type of racing (cut-point=21)

|  | Less than 21 years old (n=133) | |  |  |  | 21 or more years old (n=53) | |  |  |
| --- | --- | --- | --- | --- | --- | --- | --- | --- | --- |
|  | Male |  |  | Female |  | Male |  |  | Female |
|  | Flat (n=68) | Jump (n=37) |  | Flat (n=28) |  | Flat (n=11) | Jump (n=32) |  | Flat (n=9) |
| Spine BMD (g/cm^2^) | 0.869 ± 0.091 | 0.956 ± 0.085 |  | 0.975 ± 0.085 |  | 0.914 ± 0.089 | 0.986 ± 0.088 |  | 1.021 ± 0.067 |
| Z-Score ≤ -1.0, n (%) | 55 (82.1) | 21 (56.8) |  | 6 (21.4) |  | 5 (55.6) | 15 (50.0) |  | - |
| Z-Score ≤ -2.0, n (%) | 21 (31.3) | 5 (13.5) |  | 1 (3.6) |  | 2 (22.2) | 4 (13.3) |  | - |
| FN BMD, (g/cm^2^) | 0.828 ± 0.072 | 0.930 ± 0.100 |  | 0.832 ± 0.079 |  | 0.847 ± 0.063 | 0.942 ± 0.084 |  | 0.865 ± 0.066 |
| Z-Score ≤ -1.0, n (%) | 25 (37.3) | 5 (13.5) |  | 3 (10.7) |  | 2 (22.2) | 1 (3.3) |  | - |
| Z-Score ≤ -2.0, n (%) | 1 (1.5) | - |  | - |  | - | - |  | - |
| Total Hip BMD (g/cm^2^) | 0.921 ± 0.083 | 1.015 ± 0.115 |  | 0.954 ± 0.077 |  | 0.944 ± 0.093 | 1.020 ± 0.080 |  | 0.973 ± 0.082 |
| Z-Score ≤ -1.0, n (%) | 31 (46.3) | 6 (16.2) |  | 1 (3.6) |  | 2 (22.2) | 1 (3.3) |  | - |
